# Behavioral Inhibition in Rhesus Monkeys (*Macaca mulatta*) Is Related to the Airways Response, but Not Immune Measures, Commonly Associated with Asthma

**DOI:** 10.1371/journal.pone.0071575

**Published:** 2013-08-09

**Authors:** Katie Chun, Lisa A. Miller, Edward S. Schelegle, Dallas M. Hyde, John P. Capitanio

**Affiliations:** 1 California National Primate Research Center, University of California Davis, Davis, California, United States of America; 2 Department of Psychology, University of California Davis, Davis, California, United States of America; 3 Department of Anatomy, Physiology, and Cell Biology, University of California Davis, Davis, California, United States of America; University Hospital Freiburg, Germany

## Abstract

Behavioral inhibition reflects a disposition to react warily to novel situations, and has been associated with atopic diseases such as asthma. Retrospective work established the relationship between behavioral inhibition in rhesus monkeys (*Macaca mulatta*) and airway hyperresponsiveness, but not atopy, and the suggestion was made that behavioral inhibition might index components of asthma that are not immune-related. In the present study, we prospectively examined the relationship between behavioral inhibition and airway hyperresponsiveness, and whether hormonal and immune measures often associated with asthma were associated with behavioral inhibition and/or airway hyperresponsiveness. In a sample of 49 yearling rhesus monkeys (mean = 1.25 years, n = 24 behaviorally inhibited animals), we measured *in vitro* cytokine levels (IL-4, IL-10, IL-12, IFN-γ) in response to stimulation, as well as peripheral blood cell percentages, cortisol levels, and percentage of regulatory T-cells (CD3+CD4+CD25+FOXP3+). Airway reactivity was assessed using an inhaled methacholine challenge. Bronchoalveolar lavage was performed and the proportion of immune cells was determined. Behaviorally inhibited monkeys had airway hyperresponsiveness as indicated by the methacholine challenge (p = 0.031), confirming our earlier retrospective result. Airway hyperresponsiveness was also associated with lower lymphocyte percentages in lavage fluid and marginally lower plasma cortisol concentrations. However, none of the tested measures was significantly related to both behavioral inhibition and airway hyperresponsiveness, and so could not mediate their relationship. Airway hyperresponsiveness is common to atopic and non-atopic asthma and behavioral inhibition has been related to altered autonomic activity in other studies. Our results suggest that behavioral inhibition might index an autonomically mediated reactive airway phenotype, and that a variety of stimuli (including inflammation within lung tissue that is not specifically associated with behavioral inhibition) may trigger the airways response.

## Introduction

Asthma is a chronic disease marked by hyperresponsive airways and inflammation in the lung, and has long been recognized as having a substantial psychological component [1]. For example, behavioral inhibition (BI) in childhood, and anxiety and depression in adulthood (which can be adult manifestations of a behaviorally inhibited temperament in childhood: [2]–[5]) have been associated with asthma. These relationships have been established by comparing individuals (a) with and without asthma [6], [7], (b) with less- vs. more-severe asthma [8]–[10], and (c) over time, using prospective methodology [11].

While the previously described studies are compelling, many used a broad definition of asthma, and relied on self- or parent-reports rather than biological measures. In addition, the designs of most of these studies do not permit the understanding of whether behavioral inhibition precedes asthma, follows asthma, or if a third factor underlies both BI and asthma. Finally, nearly all of the studies investigating psychological functioning and asthma have not distinguished between two broad types of asthma (atopic vs. non-atopic). Airway hyperresponsiveness (AHR) and inflammation characterize both types of asthma; atopic (allergic) asthmatics, however, react to a known aeroallergen, which typically triggers the airways response, while non-atopic (non-allergic) asthmatics do not [12].

Understanding mechanisms of how BI might relate to asthma can be substantially enhanced through use of an animal model, and monkey models have been especially valuable in recapitulating most aspects of asthma in humans [13]–[15]. Decades of research have likewise demonstrated close similarity between humans and rhesus monkeys in behavioral and physiological measures of behavioral inhibition [16], [17]. In fact, in a recent retrospective study in our laboratory, we found that behavioral inhibition in infancy was associated with AHR in adolescent and young adult rhesus monkeys, but was not associated with atopy [18]. Airway responsiveness, commonly assessed by recording the dosage of a bronchoconstrictor needed to increase airways resistance by a fixed amount, presents a much better measure of asthma than self- or parent-reports and is commonly used to confirm a diagnosis of asthma [19].

Although our previous study showed that behavioral inhibition was associated with AHR [18], retrospective analyses run the risk of generating spurious results because of chance associations in the data; consequently, it is important to confirm these results using prospective methodology. Furthermore, our previous study did not assess potential mechanisms of how BI might relate to AHR; while BI was not related to an immediate hypersensitivity response [18], it might still be associated with the immune responses characteristic of asthma. In the present study we focused on (1) the T-helper type 2 (T_H_2) cytokine IL-4, which could promote an asthmatic phenotype through recruitment of inflammatory cells (e.g., eosinophils) to the airway [20]; T-helper type 1 (T_H_1) cytokines (IL-12, IFN-γ), which are considered protective [21]; and IL-10 associated with regulatory T-cells (T_Regs_) [22]; (2) T_Regs_ in peripheral blood, which suppress T_H_2-type responses and have been found to be higher in healthy subjects [23]; (3) concentrations of the anti-inflammatory glucocorticoid, cortisol, in peripheral blood; and (4) cell differentials in peripheral blood and lavage fluid. Our principal hypothesis was that behaviorally inhibited animals would show a significant relation to AHR. Our second hypothesis was that the relationship between BI and AHR would be mediated by asthma-relevant measures [cytokine gene expression, T_Regs_, cortisol, complete blood counts (CBCs), and bronchoalveolar lavage fluid (BALF) differential counts].

## Materials and Methods

### Subjects and Living Arrangements

Subjects were 49 gonadally-intact rhesus monkeys (*Macaca mulatta*; 24 males). All animals were born into one of 21 outdoor, half-acre, field corrals at the California National Primate Research Center (CNPRC). Corrals were constructed of chain-link sides and top, and contained a natural substrate, several wooden A-frame structures and canopy for protection from wind and rain, perches in multiple locations throughout the cage, and several other climbing structures used as enrichment. Each cage contained up to 150 animals of all age and sex classes. Animals were provided water ad libitum and fed laboratory monkey chow (Lab Diet #5038, Purina Mills International, St. Louis, MO) twice a day in the morning between 0700 and 0800 and in the afternoon between 1400 and 1500. Twice weekly, fruit and vegetable supplements were provided. In addition, trained animal care technicians checked animals’ health daily. All subjects were assessed at both infant and yearling time points.

When animals were moved indoors for the 25-hr infant assessment (see below), they were placed in an individual holding cage in the morning and provided with a cloth diaper, stuffed terry cloth duck, and a novel object. To better understand responses to relocation and maternal separation, infants were hand-caught to obtain blood samples and behavioral assessments were done in the holding cage. Infants were provided with monkey chow, water, and fruit-flavored juice and were supplemented with fruit.

For yearling assessments, animals were removed from the outdoor corral for 4 days and placed in an indoor holding cage. Animals were provided with water ad libitum and monkey chow twice daily, in the morning (between 0700 and 0800) and afternoon (between 1400 and 1500). Yearlings were provided with additional enrichment including a Nylabone Dental Chew (Nylabone, Neptune, NJ) and a mirror.

### Infant Assessments – Behavioral Inhibition

Between 93 and 124 days of age (mean = 110.2), all animals participated in CNPRC’s BioBehavioral Assessment (BBA) program, which was designed to assess animals’ behavioral and physiological responsiveness and temperament. Details are described elsewhere [24], [25]. Briefly, infants were removed from their field cages, separated from their mothers, and housed individually for a 25-hour period, during which time each infant experienced several separate assessments. At the end of this period, animals were returned to their mothers and their field corrals. We defined BI using criteria identical to that in our retrospective analysis of BI and airways response [18]: behaviorally inhibited animals were identified by low emotionality (based on focal animal observations), high vigilance (based on temperament ratings conducted at the end of the 25-hr period), and blunted cortisol response (determined seven hours after separation/relocation). Other laboratories have shown continuity of BI in both rhesus monkeys [26], [27] and humans [28]–[30] using similar measures. We selected 24 animals that were behaviorally inhibited, and the remaining 25 animals served as non-behaviorally inhibited controls.

### Yearling Assessments

When subjects were yearlings (mean = 1.25; range = 1.11 to 1.36 years), animals were briefly net-caught in their field corrals at 0900 h and again at 1500 h on the same day, and blood was sampled from the femoral vein for later assessment of plasma cortisol concentrations (see Table 1). Between 4 to 31 days later, pairs of animals (one behaviorally inhibited, one control) were relocated by 0900 h from their corrals to individual housing indoors for a four-day period. On Day 1 of the relocation, evaluations included one blood sample to assess plasma cortisol concentrations two hours after relocation (1100 h). On Day 2 of relocation at 1000 h, animals experienced blood sampling via femoral venipuncture following manual restraint; cells were used for stimulated cytokine gene expression (T_H_1/T_H_2 cytokines), complete blood count (CBC), and peripheral blood mononuclear cell (PBMC) isolation for immunophenotyping of T_Regs_. On Day 3 of relocation, the animal's lung function was assessed via airway responsive testing. On Day 4 of relocation (n = 38), bronchoalveolar lavage fluid sampling occurred. Yearling assessments and study design are shown in Table 1.

**Table 1:** Yearling assessments study design and outcome measures.

Time	Procedure
4–31 days prior to relocation	AM/PM Cortisol
*Relocation*	
Day 1	Relocation Cortisol
Day 2	CBC/PBMC/Stimulated Cytokine Gene Expression
Day 3	PFT
Day 4	BALF

CBC = complete blood count.

PBMC = peripheral blood mononuclear cell isolation.

PFT = pulmonary function test.

BALF = bronchoalveolar lavage fluid collection.

Airway responsiveness testing and BALF sampling were performed under ketamine and propofol anesthesia and efforts were made to minimize suffering of animals by having a veterinarian or clinical veterinary technician present throughout all procedures to monitor the status of the animal; procedures were terminated when oxygen saturation fell below 70% of baseline. After physiological data collection, a veterinarian or clinical veterinary technician monitored animals regularly until the animal was able to sit up. Animals’ health was checked daily during experimental procedures. No animals were sacrificed for this study; at the end of the yearling assessment period, animals were returned to their corrals. All procedures were approved by the University of California, Davis, Institutional Animal Care and Use Committee (Protocol Number: 15344) and followed the Guide to the Care and Use of Laboratory Animals of the National Institutes of Health. The University of California Davis and the CNPRC are accredited by the Association for Assessment and Accreditation of Laboratory Animal Care.

#### Plasma cortisol concentrations

At each time point where cortisol was to be measured, we centrifuged 0.5 ml of whole blood from each sample for 10 minutes at 1900 g at 4°C. Plasma was removed and decanted into tubes for storage at −80°C and was later assayed in duplicate using commercially available kits (Diagnostics Products Corporation, Los Angeles, CA). Inter- and intra- assay coefficients of variation were 5.4% and 3.4%, respectively.

#### Stimulated cytokine gene expression

Heparinized whole blood was diluted 1.25∶1 with DMEM culture media (Invitrogen, Carlsbad, CA) and stimulated with staphylococcal enterotoxin B (SEB) (Toxin Technology, Sarasota, FL) in a sterile 24-well plate. The final well concentrations were 500 ng/ml SEB. Cultures were incubated for 24-hours at 37°C in an atmosphere of 5% CO_2_, then plates were briefly centrifuged and pellets were stored in RNALater (Ambion, Austin, TX) for a time period of 4–6 months at −80°C for mRNA extraction. We quantified relative gene expression of cytokines using quantitative polymerase chain reaction (qPCR) for IL-4 (NCBI accession no. NM_001032904), IL-10 (NCBI accession no. NM_001044727), IL-12 (NCBI accession no. NM_001044725), and IFN-γ (NCBI accession no. NM_001032905).

#### Quantitative PCR

We extracted total RNA from stimulated cells with the RiboPure Blood kit (Ambion, Austin, TX) with modifications of manufacturer’s instructions (Supporting Information S1). Concentration of mRNA ranged from 10–141 ng/ul (mean = 36.13, SD = 22.63) and 260/280 values that ranged from 1.56–2.27. We synthesized cDNA using the QuantiTect Reverse Transcription kit (Qiagen, Germantown, MD) following manufacturer’s instructions. The amount of genomic DNA wipeout buffer was used appropriately to the concentration of mRNA.

We ordered qPCR primers and probes from Integrated DNA Technologies (San Diego, CA) and Applied Biosystems (Foster City, CA) respectively (Table S1). The qPCR experiments were conducted using TaqMan detection chemistry on an ABI Prism 7900 qPCR machine (Applied Biosystems, Foster City, CA). Taqman Universal Master Mix II no UNG (Applied Biosystems, Foster City, CA) was used at a final concentration of 1X. Forward and reverse primer concentrations were 0.4 µM and the probe concentration was 0.27 µM. We generated efficiency curves of primer and probe pairs from cDNA from various blood samples using four 10-fold dilutions. The efficiency of all primer probe sets was >92%. The dynamic range of the standard curves for each gene encompassed all sample quantification cycle (C_q_) values. The qPCR reaction was run at 50°C for 2 minutes, 95°C for 10 minutes, and 45 cycles of 95°C for 15 seconds and 60°C for 1 minute. Each sample was run in duplicate with standard deviations <0.5. We analyzed the qPCR results using the Applied Biosystems Sequence Detection System software (SDS v. 2.2.1), and we normalized gene expression using GAPDH (NCBI accession no. NM_001195426). In rhesus monkeys, GAPDH has been shown to be a reliable and valid reference gene [31]. For all analyses, transformed inverse delta-Ct [−ΔCt = -(C_q test gene_ – C_q normalizer_)] values were used for gene expression.

#### Immunoprofiling of regulatory T cells by flow cytometry

**Figure 1:**
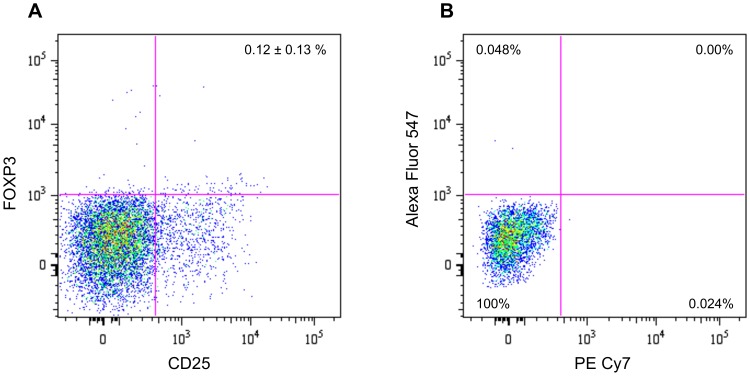
Immunoprofiling of regulatory T-cells in isolated PBMCs. (A) CD3+CD4+ lymphocytes evaluated for CD25, FOXP3; (B) CD3+ CD4+ isotype controls for PE Cy7 and Alexa Fluor 647. Mean and standard deviation of total PBMCs are presented for (A), while parent CD3+CD4+ percentages are presented for (B).

We isolated peripheral blood mononuclear cells using a Ficoll density gradient (Histopaque 1077, Sigma, St Louis, MO), then cells were washed twice with Hanks’ modified medium. Cells were cryopreserved in 90% fetal bovine serum (FBS) and 10% DMSO for later immunophenotyping by resuspending (1 to 2×10^5^ cells per sample) in PBS as previously described [32]. Immunostaining for lymphocyte characterization used the following panel to look at the T_Reg_ population [33]: mouse monoclonal anti-human CD3 (pacific blue), CD4 (Per CP-Cy5.5), CD25 (PE Cy7), FOXP3 (Alexa Fluor 647) (BD Biosciences, Sparks, MD) (Figure 1). Two- and three-color analysis was performed on a FACS Aria II (BD Biosciences, Sparks, MD) flow cytometer, acquiring 40,000–60,000 events per sample and analyzed with Flow Jo software (Tree Star, Inc., Ashland, OR).

#### Complete blood count

Blood was drawn into an EDTA tube and a complete blood count was performed by the CNPRC’s Clinical Laboratory using a Serono Baker Diagnostic System (Allentown, PA). All electronic counts were verified by a manual differential. Values were expressed as percentages to be consistent with presentation of BALF data.

#### Airway responsiveness testing

Airway responsiveness to doubling concentrations of inhaled methacholine aerosols was quantified using previously published procedures [14], [18]. Each animal was anesthetized with ketamine (10 mg/kg) and anesthesia was maintained with propofol (0.1 mg/kg/min). Animals were intubated and placed in a plethysmograph with the intubation tube attached to a pneumotachograph assembly. Airways resistance was assessed using a forced oscillatory technique and a dose-response curve was generated for each individual [14]. Methacholine was administered as an aerosol at a set tidal volume and breathing frequency (15.0 ml/kg and 30.0 bpm) using a compressed air nebulizer in series with a positive pressure ventilator. We used repeated 30-sec challenge periods separated by 240-sec data collection periods, beginning with saline followed by doubling concentrations of methacholine starting at 0.0625 mg/ml and ending at a concentration that increases airways resistance to 200% of baseline (EC200), or a concentration of 32.00 mg/ml unless a monkey's oxygen saturation went below 70.0% or less. Animals with an EC200 of 8 mg/ml methacholine or less were considered to have AHR. This cutoff is similar to values used to define AHR in human asthmatics [34], [35] and allergen sensitized and challenged rhesus monkeys [36]. Testing was terminated at 32 mg/ml methacholine because this concentration is well above the concentration needed to identify AHR and also reduces the likelihood inducing systemic effects, including increases in heart rate and blood pressure and altered vascular perfusion. In our sample, 20 animals had responses from 0.31–8.00 mg/ml and were designated hyperresponders, while 29 animals ranged from 10.91–32.00 mg/ml and were considered normal responders.

#### Bronchoalveolar lavage

Animals were anesthetized with ketamine (10 mg/kg) and anesthesia was maintained with propofol (0.1 mg/kg/min). As described previously [14], 10 ml of endotoxin-free PBS (Sigma, St. Louis, MO) was instilled through a bronchoscope. A single technician performed all lavage cell differentials on each sample twice and averages from the two differentials were used for analysis. Lavage samples were cytocentrifuged, air dried, stained with a modified Wright’s stain, and proportion of macrophages, neutrophils, eosinophils, lymphocytes, and epithelial cells were determined by counting approximately 300 cells/subject by light microscopy.

### Statistical Analyses

To test our hypotheses that behaviorally inhibited animals would show a significant relation to AHR and that this relationship would be mediated by asthma-relevant measures, we intended to use a mediation model based on Baron and Kenny [37]. After demonstrating a relationship between AHR and BI (using a Chi-Square test), the next step in a mediational analysis is to compute Pearson correlation coefficients between BI and the possible mediators, and between AHR and the possible mediators. The relationship between AHR and BI was tested using a one-tailed test based on our directional hypothesis from our retrospective study [18]. To continue a mediational analysis, the mediator must show a significant relationship with both the independent variable (BI) and the dependent variable (AHR); this did not occur, and so the full mediational analysis could not be performed; consequently only the bivariate relationships are reported. All statistical analyses were performed with SPSS 18.0 (IBM Corporation, Armank, NY). All variables were transformed (log, root, power, or reciprocal) as needed to achieve normality.

## Results

### Airway Responsiveness

**Figure 2:**
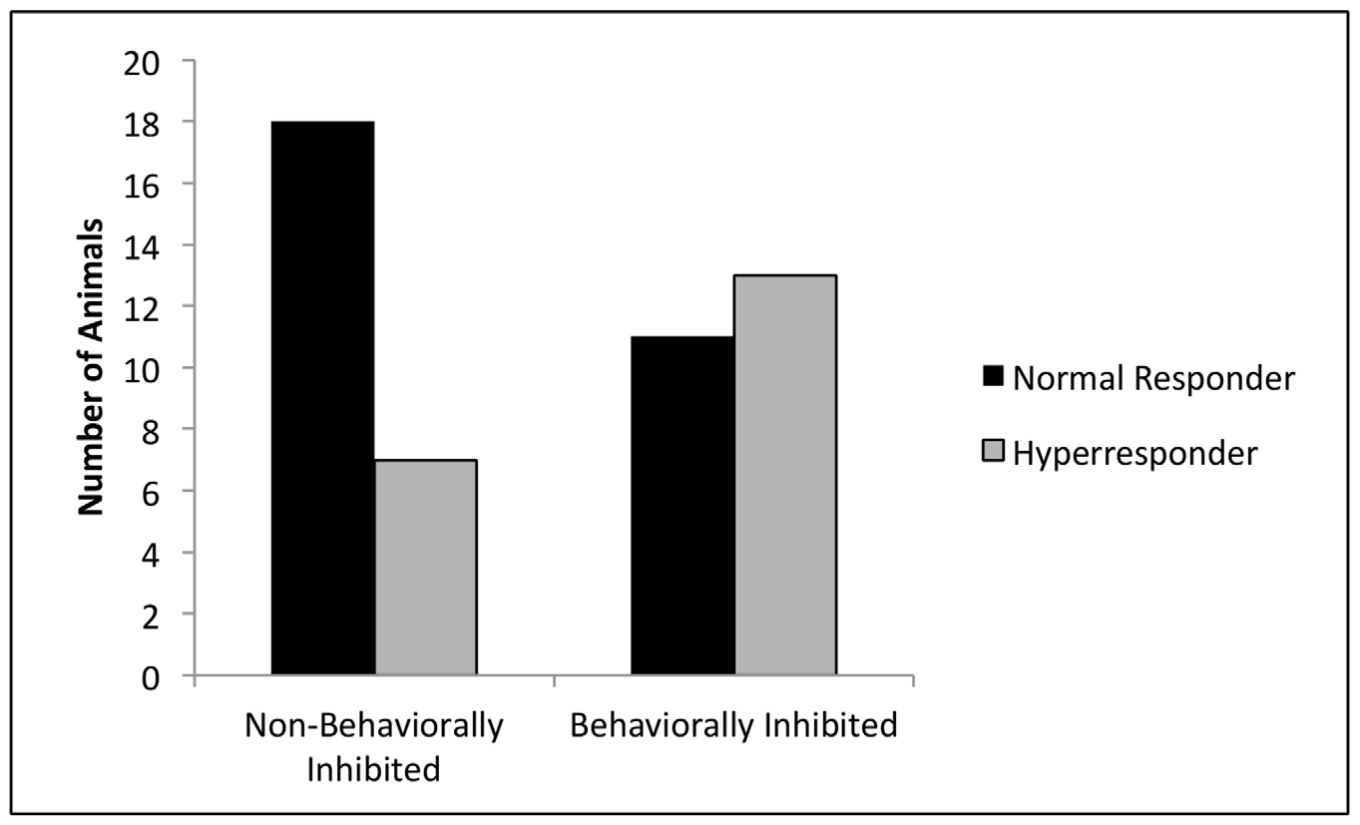
Comparison of the number of subjects who are normal responders and hyperresponders in non-behaviorally inhibited and behaviorally inhibited groups.

Behaviorally inhibited animals were more likely to be hyperresponders compared to control animals (*Chi-Square* = 3.471, p = 0.031) (Figure 2). Raw values for EC200 are shown in Table S2.

### Correlations of BI and Hyperresponsiveness with Potential Mediators

Bivariate analyses revealed that sex was associated with percentage of eosinophils in lavage fluid (p = .054), age was associated with IFN-γ gene expression (p = .049) and sample collection time was related to the AM outdoor cortisol value (r = .581, p<.001); consequently, partial correlations are reported for these measures. All other variables showed no effects of age and sex (p>.102), thus Pearson correlations are presented. Several correlation coefficients between BI/AHR and the potential mediating variables were significant (Table 2); however, none of the potential mediators was significantly associated with both BI and AHR, a necessary requirement for a full mediational analysis. Consequently, we discuss bivariate relationships among variables next.

**Table 2:** Correlation coefficients between physiological measures, behavioral inhibition, and AHR.

Mediators		Behavioral Inhibition	AHR
Gene Expression (n = 49)	IL-4 gene expression	−0.001	0.087
	IL-10 gene expression	0.180	0.066
	IL-12 gene expression	−0.179	−0.197
	IFN-γ gene expression	−0.293* ^†^	−0.084^†^
Plasma Cortisol Levels (n = 49)	Natal Cage AM	−0.235^†^	−0.254+^†^
	Natal Cage PM	−0.122	−0.159
	Relocation Cortisol	−0.146	−0.124
Lavage Percentages (n = 38)	Macrophages/Monocytes	−0.051	−0.030
	Lymphocytes	−0.191	−0.351*
	Neutrophils	0.147	0.176
	Eosinophils	−0.294+^†^	−0.253^†^
Blood Percentages (n = 49)	Neutrophils	0.016	−0.155
	Lymphocytes	−0.065	0.083
	Monocytes	0.102	0.239+
	Eosinophils	0.329*	0.166
Regulatory T cells (n = 49)	Regulatory T cells	0.183	0.022

Values expressed are Pearson product-moment correlation coefficients, unless designated by ^†^, which reflect partial correlation coefficients (see text).

* p<0.05.

+p<0.10.

AHR = Airway hyperresponsiveness.

IL = Interleukin.

IFN = Interferon.

#### T_H_1/T_H_2 cytokines

Behaviorally inhibited animals had lower levels of SEB-stimulated IFN-γ expression (partial correlation [pr] = –0.293, p = .043) (Table 2). Gene expression levels for the three other cytokines (IL-4, IL-10, and IL-12) were unrelated to either BI or AHR. Descriptive statistics of means and standard deviations of −ΔCt values are included in Table 3.

**Table 3:** Gene expression, lavage differentials, blood differentials, and regulatory T-cell percentage means and standard deviations.

Mediators		Behaviorally Inhibited ( 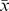 ± *s*)	Non-Behaviorally Inhibited ( 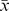 ± *s*)
Gene Expression (n = 49)	IL-4 gene expression (−ΔCt)	−10.75±3.25	−10.63±3.05
	IL-10 gene expression (−ΔCt)	−9.42±3.09	−8.84±3.17
	IL-12 gene expression (−ΔCt)	−14.66±3.45	−13.48±3.34
	IFN-γ gene expression (−ΔCt)	−2.86±2.39	−1.98±2.17
Lavage Percentages (n = 38)	Macrophages/Monocytes	80.09±17.35	83.63±7.64
	Lymphocytes	8.67±3.78	9.95±3.63
	Neutrophils	10.77±18.10	5.08±6.09
	Eosinophils	0.47±0.53	1.34±1.86
Blood Percentages (n = 49)	Neutrophils	64.13±11.09	63.96±10.13
	Lymphocytes	31.67±10.66	32.68±9.78
	Monocytes	2.50±1.67	2.52±2.37
	Eosinophils	1.71±1.78	0.80±1.08
	Regulatory T cells (% PBMCs)	0.12±0.10	0.12±0.15

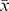
± s = mean ± standard deviation.

PBMCs = Peripheral Blood Mononuclear Cells.

Note: ”−ΔCt” values are shown because lower values correspond with lower levels of gene expression, while higher values correlate with higher levels of expression.

#### Plasma cortisol concentrations

Hyperresponsive animals had marginally lower cortisol concentrations in the natal cage at the AM time point (pr = –.254, p = .082) (Table 2).

#### Cell differentials (lavage fluid and peripheral blood)

Individuals with AHR had a lower percentage of lymphocytes (r = –.351, p = .031) in lavage fluid and a marginally higher percentage of blood monocytes (r = .239, p = .099). Behaviorally inhibited animals showed a trend toward a lower percentage of eosinophils in BALF (pr = –.294, p = .078) and a significantly higher percentage of blood eosinophils (r = 0.329, p = .021) (Table 2). Descriptive statistics of means and standard deviations of cell differential counts (BALF and blood) are included in Table 3.

## Discussion

Animals identified in infancy as behaviorally inhibited (indexed by measures of reduced emotionality, greater vigilance, and blunted cortisol response in stressful circumstances) were significantly more likely, compared to controls, to display airways hyperresponsiveness (measured by a methacholine challenge) approximately one year later, confirming our previous retrospective results [18]. We assessed potential neuroendocrine and immune mediators of the relationship between BI and AHR by focusing on T_H_1/T_H_2 cytokines, T_Regs_, plasma cortisol concentrations, and cell differentials (blood and BALF), and found that, although there were some significant relationships between the potential mediators and either BI or AHR, none of the tested measures was significantly related to both BI and AHR; consequently, a mediational analysis could not be performed. While we recognize that our list of possible mediators was not exhaustive, we believe our data provide further evidence that the role of temperament in asthma might be unrelated to the immune component of the airways response.

### Behavioral Inhibition and the Airways Response

Consistent with our earlier retrospective study [18], we found that BI was associated with more reactive airways. As described above, behavioral inhibition in childhood (as well as anxiety and depression in adulthood, which can be mature consequences of BI in childhood) has been related to various measures of asthma in numerous studies [6]–[11]. The present results, along with our earlier one, indicate that the rhesus monkey provides a robust model system in which to explore mechanisms of this relationship, where biobehavioral measures quantified during infancy may identify animals at risk for developing hyperresponsive airways. This model could also provide translational opportunities to better understand the development of asthma.

### Correlations of BI and AHR with Possible Mediators

While we found some significant (and marginally significant) correlations between our measures of BI and AHR on the one hand and potential neuroendocrine and immune mediators on the other hand, there were no neuroendocrine or immune measures that were related to both BI and AHR; consequently, we could not identify any mediators of the BI-AHR relationship. We recognize that we did not test all possible mediators and that future research may identify such a relationship. Here, we discuss the relationships that were found.

Asthma has been commonly characterized by a T_H_2 disposition [38], and our expectation was that both behaviorally inhibited and hyperresponsive animals might show increased expression of T_H_2 cytokines and decreased expression of T_H_1 cytokines. We did find that behaviorally inhibited animals showed decreased IFN-γ (T_H_1 cytokine) expression, but IFN-γ expression was unrelated to AHR. In addition, IL-10 has been thought to have a role in effective suppression of allergic responses of the lung and is a utilized pathway of suppression by T_Regs_ [39]; our failure to find relationships between these cytokines and AHR suggests that airway responsiveness may not be related to a simple T_H_1/T_H_2 explanation.

Asthmatics and individuals with atopic disorders have been shown to have attenuated cortisol concentrations when compared to controls [40]. Consistent with this result, we found that animals with more responsive airways had lower levels of AM cortisol (a trend) (Table 2), which, if confirmed, could suggest dysregulation of the HPA axis. Altered regulation of the HPA axis may contribute to the progression of inflammatory disease, where glucocorticoids have been found to shift the immune response from a T_H_1 to a T_H_2 response [41]. We note that a blunted cortisol response was one of our infant measures used to identify behavioral inhibition and because cortisol has trait-like properties [42], [43], cortisol may be a useful biomarker for identification of individuals at risk for developing AHR.

We found that hyperresponders showed a lower percentage of lymphocytes in lavage fluid and a marginally higher percentage of monocytes in blood (Table 2). Asthma is typically characterized by infiltration into lung tissue by a wide range of cells including eosinophils, basophilic cells, and activated T cells [44], [45]. However, measures in BALF and blood are not necessarily reflective of tissue measures [45], and we suggest that further research should examine other measures of inflammation within lung tissue. It would be helpful to phenotype these cells, inasmuch as others have reported that asthmatics have higher numbers of T_H_2-like T-lymphocytes in BALF, even in the absence of differences in overall numbers of lymphocytes [46]. Unfortunately, we recovered too few cells in BALF in the current study to perform such phenotyping.

Few studies have examined how BI relates to immune function. Behaviorally inhibited rhesus monkeys show reduced measures of cellular immunity (reduced lymphocyte proliferation to mitogens) compared to controls [47]–[49]. Our findings that behaviorally inhibited animals had lower levels of IFN-γ are consistent with this idea: lower levels of IFN-γ may indicate reduced cellular immunity. However, we are aware of no studies of BI that have examined asthma-relevant immune measures. We found that behaviorally inhibited animals had a higher percentage of blood eosinophils as well as a trend toward a lower percentage of eosinophils in lavage fluid (Table 2). These results may be indicative of alterations in eosinophil trafficking in behaviorally inhibited individuals. We note, however, that asthma is commonly associated with higher numbers of eosinophils in blood and lavage fluid [50], [51], a result that we did not find for our most relevant asthma measure, AHR. Although BI was related to this asthma-relevant measure, our analysis could not demonstrate that any immune measure mediated the relationship between BI and AHR.

Overall, we believe these results provide a new focus for future studies, and suggest that the role of temperament in asthma may be unrelated to the immune component of asthma; rather BI and AHR may both be indicators of a particular central and autonomic nervous system organization. A common feature of both BI and AHR is altered autonomic reactivity: behaviorally inhibited children have shown alterations in heart period compared to non-behaviorally inhibited children [52], and compelling evidence exists showing that autonomic dysfunction and/or dysregulation contributes to hyperresponsive airways [53]. Centrally, BI has been associated with altered responsiveness of the amygdala and hippocampus [54], and the later psychosocial manifestations of BI are successfully treated with drugs targeting brain monoamine systems [55]. Lung function has been associated with circulating serotonin levels, where higher levels of serotonin were related to decreased lung function [56], [57] and treatment that decreased serotonin plasma levels led to increased lung function [58]. Airway smooth muscle contraction is modulated by serotonin both through direct stimulation and activation of parasympathetic nerve fibers innervating airway smooth muscle. Dysregulation of serotonin signaling in parasympathetic ganglia innervating airway smooth muscle may lead to AHR [59]. It is possible that hyper-sensitive airways, resulting from autonomic dysregulation, could be activated by a variety of triggers, including aeroallergens, as is seen in atopic asthma, or by unknown factors, as seen in non-atopic asthma. We believe an investigation of neural control within lung tissue may contribute to our understanding of BI and AHR and bears further investigation.

### Conclusions

Asthma has long been associated with psychological functioning, and the current study used a prospective methodology in an animal model to relate BI to the airways response. The present study did not elucidate the mechanism by which AHR and BI are related. However, we believe that the monkey model could be valuable in further understanding the development of AHR in behaviorally inhibited individuals from infancy by investigating simultaneously autonomic function and neural control of lung tissue. Should such a relationship be established, this model could be very useful for developing interventions to prevent the development of AHR.

## Supporting Information

Table S1 Primer and probe sequences.
(XLSX)

Table S2 Raw EC200 values for each animal.
(XLSX)

Supporting Information S1 List of modifications to RiboPure - blood procedure.
(DOC)
