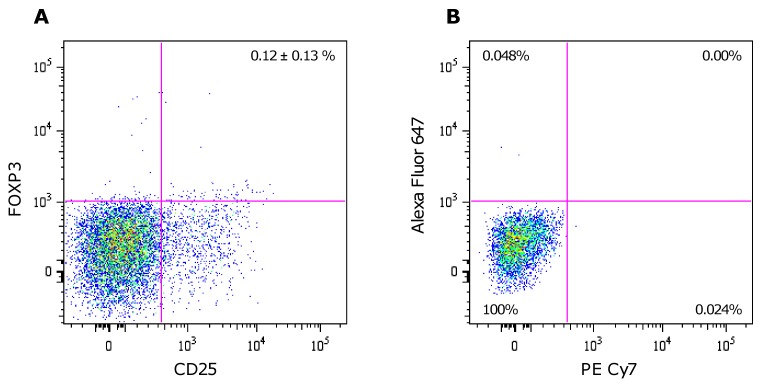# Correction: Behavioral Inhibition in Rhesus Monkeys (*Macaca mulatta*) Is Related to the Airways Response, but Not Immune Measures, Commonly Associated with Asthma

**DOI:** 10.1371/annotation/aade3c83-f488-446e-8b02-bca381d2fe60

**Published:** 2013-08-20

**Authors:** Katie Chun, Lisa A. Miller, Edward S. Schelegle, Dallas M. Hyde, John P. Capitanio

There was an error in Figure 1. The correct version of the figure is available here: 

**Figure:**