# Supplementary material for: Behavioral Inhibition in Rhesus Monkeys (Macaca mulatta) Is Related to the Airways Response, but Not Immune Measures, Commonly Associated with Asthma
Source: PLoS One. 2013 Aug 9;8(8):e71575. doi: 10.1371/journal.pone.0071575 (PMC3739724; doi:10.1371/journal.pone.0071575)
Supplement: Supporting Information S1 — List of modifications to RiboPure - blood procedure. (DOC) [file pone.0071575.s003.doc]

Appendix S1.

List of Modifications to RiboPure - Blood Procedure

1. After removal of supernatant after addition of acid-phenol, incubated at room temperature for 10 minutes, not 5 minutes. Next, centrifuged for 5 minutes, not 1 minute.
2. After column was washed the second time with Wash Solution 2/3, centrifuged for 2 minutes, not 1 minute.
3. Messenger RNA was eluted with 30 ul of elution solution, not 50 ul. Elution solution was also heated to 95° C, not 75° C.
4. Elution solution was incubated for 2 minutes on column, not 20 seconds.
